# Is the eye a window to the brain in Sanfilippo syndrome?

**DOI:** 10.1186/s40478-020-01070-w

**Published:** 2020-11-17

**Authors:** Helen Beard, Glyn Chidlow, Daniel Neumann, Nazzmer Nazri, Meghan Douglass, Paul J. Trim, Marten F. Snel, Robert J. Casson, Kim M. Hemsley

**Affiliations:** 1 Childhood Dementia Research Group, Hopwood Centre for Neurobiology, Lifelong Health Theme, South Australian Health and Medical Research Institute, Adelaide, SA Australia; 2 Present Address: Childhood Dementia Research Group, College of Medicine and Public Health, Finders Health and Medical Research Institute (FHMRI), Flinders University, Bedford Park, SA Australia; 3 Ophthalmic Research Laboratories, Discipline of Ophthalmology and Visual Sciences, University of Adelaide, Adelaide, SA Australia; 4 Proteomics, Metabolomics and MS-Imaging Facility, South Australian Health and Medical Research Institute, Adelaide, SA Australia

**Keywords:** Retina, Mucopolysaccharidosis type III, Central nervous system, Eye, Lysosomal

## Abstract

Sanfilippo syndrome is an untreatable form of childhood-onset dementia. Whilst several therapeutic strategies are being evaluated in human clinical trials including i.v. delivery of AAV9-based gene therapy, an urgent unmet need is the availability of non-invasive, quantitative measures of neurodegeneration. We hypothesise that as part of the central nervous system, the retina may provide a window through which to ‘visualise’ degenerative lesions in brain and amelioration of them following treatment. This is reliant on the age of onset and the rate of disease progression being equivalent in retina and brain. For the first time we have assessed in parallel, the nature, age of onset and rate of retinal and brain degeneration in a mouse model of Sanfilippo syndrome. Significant accumulation of heparan sulphate and expansion of the endo/lysosomal system was observed in both retina and brain pre-symptomatically (by 3 weeks of age). Robust and early activation of micro- and macroglia was also observed in both tissues. There was substantial thinning of retina and loss of rod and cone photoreceptors by ~ 12 weeks of age, a time at which cognitive symptoms are noted. Intravenous delivery of a clinically relevant AAV9-human sulphamidase vector to neonatal mice prevented disease lesion appearance in retina and most areas of brain when assessed 6 weeks later. Collectively, the findings highlight the previously unrecognised early and significant involvement of retina in the Sanfilippo disease process, lesions that are preventable by neonatal treatment with AAV9-sulphamidase. Critically, our data demonstrate for the first time that the advancement of retinal disease parallels that occurring in brain in Sanfilippo syndrome, thus retina may provide an easily accessible neural tissue via which brain disease development and its amelioration with treatment can be monitored.

**Electronic supplementary material:**

The online version of this article (10.1186/s40478-020-01070-w) contains supplementary material, which is available to authorized users.

## Introduction

The temporal course of neurodegeneration in Sanfilippo syndrome or mucopolysaccharidosis type IIIA (MPS IIIA) is manifest by a normal infancy followed by progressive intellectual disability in early childhood and death at a median age of 18 years [43]. The most common of four subtypes of Sanfilippo syndrome in Australia [28], MPS IIIA results from an inherited, recessive mutation in the gene encoding the lysosomal exoenzyme sulphamidase (SGSH; EC 3.10.1.1), leading to incomplete degradation of heparan sulphate (HS), its accumulation in lysosomes and initiation of an inflammatory and degenerative cascade predominantly affecting the central nervous system (CNS).

Sanfilippo syndrome is presently untreatable, however several therapeutic approaches are under clinical evaluation in patients including but not limited to gene replacement [11, 19, 40]. The monitoring of both disease progression and therapeutic efficacy however, remains a significant obstacle and there is an urgent unmet need for practical, non-invasive, and widely available techniques to be developed.

Significant retinal pathology occurs in utero in MPS III [6] with inclusions observed in photoreceptor cells, retinal ganglion cells and glia in the optic nerve. The electroretinogram (ERG) becomes abnormal in childhood [3, 9, 13, 27]. Gills and colleagues [13] reported retinal pigmentation, impairment of the ERG and night-blindness in two MPS III patients. In post-mortem specimens from adults with MPS III, vacuolation of the retina is observed, with photoreceptor degeneration. Haer-Wigman et al. [16] found mutations in the gene encoding the lysosomal enzyme defective in MPS IIIC (heparan acetyl-CoA: alpha-glucosaminide N-acetyltransferase) in six adults with retinitis pigmentosa. Typical cognitive decline was not a clinical feature. This finding was recently verified in a cohort of 17 adults by Schiff and colleagues [35]. Coupled with the observation that retinal dysfunction is a common symptom in cognitively normal patients with later-onset MPS IIIA [29], these findings suggest that the retina requires a high level of lysosomal enzymes for normal function.

A significant loss of retinal function has been reported in MPS III mice as they age [18, 21, 42]. Of note, impairments to the dark-adapted ERG are evident, presumptively due to a loss of rod function, with lysosomal storage observed in Müller cells, disruption of the retinal pigment epithelium apparent from 4 weeks of age onwards, and subsequent photoreceptor degeneration. Tse et al. [42] reported that cone degeneration occurs much later in these mice (by 46 weeks of age). Emus with MPS IIIB have also been reported to exhibit vacuolisation of retinal ganglion cells [31].

Embryologically, the retina and optic nerve are outpouchings of brain and are part of CNS. Importantly, the retina is the only part of the CNS that can be visualised non-invasively. Given that many other neurological disorders exhibit retinal/optic nerve pathology prior to, or in conjunction with the onset of brain pathology [1, 24], we hypothesised that modern retinal and optic nerve imaging modalities may serve as an ideal biomarker both of progressive CNS disease in Sanfilippo syndrome and, potentially, therapeutic efficacy. The aim of the current study was to compare the temporal profiles of retinal and brain pathology and in short-term studies, determine the ability of a clinically relevant i.v.-delivered gene therapy approach to mediate improvements in disease sequelae in both neural tissues.

## Materials and methods

### Approvals

The research protocol was approved by the Institutional Animal Ethics Committee (approvals #1060/12/19 and #1109/12/21) and the Institutional Biosafety Committee (#B144/12/20 and #B149-12-21) prior to study commencement.

### Mice

Congenic MPS IIIA mice (B6.Cg-Sgsh^mps3a^) were obtained from a breeding colony established and maintained at the Women’s and Children’s Hospital Network, North Adelaide, South Australia, Australia. Congenic MPS IIIA mice were also intercrossed with a reporter line (*Thy1*-GFP M-line; [10]), purchased from Jax Mice to create MPS IIIA mice in which some retinal ganglion cells express green fluorescent protein (GFP).

All mice were group-housed in a constant temperature/humidity-controlled facility with a 14-h light:10-h dark cycle, with food and water ad libitum. Mice were provided with toilet rolls and plastic cups plus nesting material for environmental enrichment. All breeding, housing and experimental procedures complied with the Australian code for the care and use of animals for scientific purposes (8th edition; 2013) and the Association for Research in Vision and Ophthalmology (ARVO) statement (2016).

Genotyping for the *SGSH* mutation was undertaken on ear-notch tissue from which DNA was extracted (ThermoFisher Scientific), with subsequent PCR and restriction digest analysis performed according to methods in [26]. The genotypes of the GFP + mice were determined using customised Taqman® XFP genotyping assays (Thermofisher Scientific). Forward (5′ GCA CCA CCG GCA AGC T 3′) and reverse primers (5′ AGT CGT GCT GCT TCA TGT GGT 3′) and allele-specific probes (5′ CCA CC**C** TGA CCT ACG 3′) were utilised. Reactions containing 2 × TaqMan® Genotyping Mastermix and 20 × GFP-FAM primer were aliquoted into MicroAmp® fast optical 384-well reaction plates and 1 µL DNA was added to each well (5 µL total volume per well). Negative, no DNA template controls were included in each assay. The plates were sealed with MicroAmp® optical adhesive film and amplification performed on a QuantStudio 7 Flex Real-Time PCR system (Thermofisher Scientifc).

### Post-mortem extraction of tissues

No differences have previously been found in the time of onset or rate of disease lesion progression in the brains of male or female mice with MPS IIIA, therefore, mice of mixed genders were humanely killed at 3, 6, 12, 15, 20, 22 or 25 weeks of age using asphyxiation with slow**-**fill CO_2_. Intra-cardiac perfusion with ice-cold saline was undertaken then brain, eyes and optic nerves were removed. Some brain tissue was dissected into five 2 mm hemi**-**coronal slices, with slice 1 containing olfactory bulb and the rostral aspect of the cerebral cortex and slice 5 containing cerebellum and brainstem. For mass spectrometry evaluation, the retina was carefully dissected from the eyes and both brain and retina were frozen at − 80 °C until used.

Brain tissue destined for immunohistochemistry was immersion-fixed in 4% paraformaldehyde in PBS for up to 1 week. Eyes and optic nerves to be used for immunohistochemistry were immersion-fixed in Davidson’s solution (2-parts 37% formaldehyde, 3-parts 100% ethanol, 1-part glacial acetic acid and 3-parts water) for 24 h and then held in 70% alcohol for processing. Davidson’s solution is the preferred fixative for whole eyes as it provides optimal tissue morphology while avoiding retinal detachment. A separate cohort of eyes was collected for retinal whole-mount preparation and was fixed in 4% PFA in PBS for 24 h before being placed in 30% sucrose at 4 °C until dissected as previously described [8].

To determine the impact of sulphamidase gene replacement on retinal and brain disease lesions, on the day of birth, cohorts of MPS IIIA and unaffected mice (n = 5/group) received an intravenous injection of AAV9-CMV-hSGSH or AAV9-CMV-GFP (5 × 10^11^ vg; 10 µL) via the superficial temporal vein. A repeat injection was made via the tail vein on day 5 of life. Control mice of both genotypes received AAV-CMV-mCherry at this time. This sequential injection strategy increases the number of (newly emerging) retinal neurons exposed to viral vector [5]. Mice were weaned into same-sex groups at 3 weeks of age and were humanely killed as above at 6 weeks of age. Brain and both eyes were removed, and half of the brain and one eye was immersion-fixed in 4% paraformaldehyde and Davidson’s fixative, respectively, as above. Prior to being frozen at − 80 °C, the remaining brain hemisphere was divided into five 2 mm slices as above.

### Histology and immunohistochemistry

Tissues were processed routinely into paraffin with brains and eyes embedded sagittally, and optic nerves longitudinally. Six micron-thick tissue sections were cut using a rotary microtome (Leica, Wetzlar, Germany) and mounted on glass slides (Superfrost™ Plus, Thermo Scientific, USA). Retinae were sectioned in a consistent manner to the level of the optic nerve head and brain sections were taken at approximately 0.48 mm lateral to the midline (according to the Mouse Brain Atlas [32]). Oven-dried sections were deparaffinised in xylene, rinsed in two changes of 100% ethanol and gently washed in water. Haematoxylin and eosin staining was performed using standard methods.

Details of tissue type, antigen retrieval pre-treatment, primary antibody/lectin reagents used are outlined in Table 1. Biotinylated species-specific secondary antibodies, 1:2000 (Jackson ImmunoResearch Labs), Vectastain Elite ABC kit reagents (PK-6100; Vector Laboratories, CA, USA) and the diaminobenzidine (DAB) liquid substrate chromagen system (#3468; Dako, Glostrup Denmark) were used to amplify and provide chromogenic visualisation.

**Table 1:** Details of primary antibodies used for immunohistochemistry

Primary antibody/Lectin	Catalogue number, Source	Dilution	Tissue type	Pre-treatment
Peanut agglutin lectin (PNA), biotinylated	#B-1075, Vector Laboratories, California, USA	1:3000	Retina, paraffin sections	20-min microwave, 10 mM citrate buffer, pH 6
Mouse anti-rhodopsin	#MAB5316; Sigma-Aldrich Pty. Ltd. NSW, Australia	1:600	Retina, paraffin sections	15-min microwave, 10 mM citrate,2 mM EDTA, 0.5% Tween 20, pH 6
Rabbit anti-RNA-binding protein with multiple splicing (RBPMS),	#ab194213; abcam, Victoria, Australia	1:250	Retina, flat mounts	None
Goat anti-calretinin	#AF5065; R&D Systems Minneapolis, MN, USA	1:1500	Retina, paraffin sections	1 mM EDTA, 0.05% Tween 20, pH8.0 15-min, microwave
Mouse anti-protein kinase C-alpha (PKC-α)	#ab31; abcam, Victoria, Australia	1:2000	Retina, paraffin sections	1 mM EDTA, 0.05% Tween 20, pH8.0 15-min, microwave
Mouse anti-calbindin	#C9848; Sigma-Aldrich Pty. Ltd. NSW, Australia	1:1000	Retina, paraffin sections	1 mM EDTA, 0.05% Tween 20, pH8.0 15-min, microwave
Mouse anti-lysosomal integral membrane protein 2 (LIMP2)	In-house; Hemsley et. al., 2008	1:500	Retina, paraffin sections	1 mM EDTA, 0.05% Tween 20, pH8.0 15-min, microwave
		1:800	Brain, paraffin sections	Target Retrieval Solution (DAKO), 10- min microwave
Rabbit anti-glial gibrillary acidic protein (GFAP)	#Z033401-2; Agilent (DAKO), Santa Clara, CA, USA	1:500	Retina, paraffin sections	1 mM EDTA, 0.05% Tween 20, pH8.0 15-min, microwave
		1:800	Brain, paraffin sections	10-min microwave, 10 mM citrate buffer, pH 6
Isolectin-B4-peroxidase conjugated	#L5391; Sigma-Aldrich Pty. Ltd. NSW, Australia	1:60	Brain and retina, paraffin sections	0.05% Trypsin, pH 7.6 15 min @ 37 °C

Immunohistochemical staining of brain and ocular tissues was performed according to established methods [4, 7]. Briefly, both eye and brain sections were deparaffinized and specific antigen retrieval performed prior to blocking of non-specific labelling with 10% normal donkey serum (NDS) in PBS. Sections were incubated overnight in primary antibody diluted in 2% NDS, washed in PBS and endogenous peroxidases blocked in 0.3% hydrogen peroxide. Sections were then incubated at room temperature in species-specific biotinylated secondary antibody, followed by Vectastain ABC reagent and colour detection was achieved by applying DAB.

For lectin histochemistry, sections underwent antigen retrieval and then were incubated in hydrogen peroxide followed by peroxidase-conjugated isolectin-B4 overnight. Colour detection was achieved using DAB. Terminal deoxynucleotidyl transferase dUTP nick end labelling (TUNEL) was carried out according to published methods [12, 33]. All staining was batched, and all analyses were conducted by a user blinded to mouse age, genotype, and treatment status. Sections were viewed on either an Olympus BX41 (with an Olympus UC50 camera), a BX61 microscope (with a ColourView III camera) or a Leica SP8X spectral scanning confocal microscope.

### Quantification of retinal thickness

H&E-stained sections were evaluated to determine the total thickness of the retina and that of individual retinal layers. To ensure inter-animal consistency, measurements were taken in two locations: 500 µm from the ciliary body (representing “peripheral” retina) and 500 µm from the optic nerve head (representing “central” retina). Measurements were made on each side of the optic nerve head and the mean thickness of the peripheral and central retina was calculated. For accuracy, retinal thickness measurements were only taken of sections in a perpendicular orientation. Total thickness was measured from the retinal ganglion cell layer (RGC) to, but not including, the retinal pigmented epithelium (RPE). Other measurements taken were the thickness of the inner plexiform layer (IPL), inner nuclear layer (INL), outer plexiform layer (OPL), outer nuclear layer (ONL), total photoreceptor segment layer (PS), inner segment layer (IS) and outer segment layer (OS). All measurements were carried out blind to genotype/age.

### Quantification of TUNEL and immunohistochemical labelling

The number of positively stained cells in a specified length of retina or in a given area of brain were either counted manually or quantified using threshold analysis. The relatively low number of TUNEL-positive nuclei were counted along the entire length and thickness of retinae. Peanut agglutinin-positive cone inner segment number were counted in a field of view using a 40X objective on each side of the central and peripheral retina. Calretinin positive amacrine cells were counted along 450 µm of retina starting 150 µm from each side of the optic nerve head. PKCα-positive bipolar cells were counted in the central and peripheral retina, for a total length of ~ 225 µm on both sides of the optic nerve head. Due to their relatively low number, calbindin-positive horizontal cells were counted along almost the entire length of retina, starting one field of view from the ciliary body to within one field of view from the optic nerve head using a 40X objective. Cell counts were expressed as number of immuno-positive cells/mm. Threshold analysis of LIMP2 and GFAP staining, based on the optical density of positive immunostaining, is reported as % immunoreactivity. Rhodopsin staining was undertaken for demonstration of rod outer segment changes however no quantification was performed.

### Preparation of flat mounts for evaluation of RGC number and dendritic arbour

Immunolabelling with RBPMS was carried out on free-floating retinae, which were incubated in primary antibody for 3 days at room temperature, washed in PBS with 1% triton**-**X and then incubated in Alexafluor 488-labelled anti-rabbit secondary antibody for 1 day at 4 °C. Following further washing in PBS/triton**-**X, retinae were mounted on glass slides using Vectashield anti-fade mounting medium with DAPI (#H-1200, Vector Laboratories, Burlingame, USA) and stored in the dark. Flat mounts were evaluated on a confocal microscope (SP8X, Leica, Germany) with excitation provided by a 488 nm laser with emission captured with 493–569 nm filters for the green channel.

The density of RGCs was quantified in retinas taken from n = 6 mice/genotype at each of 3, 6, 12 and 20 weeks of age. A further n = 3 mice/genotype were aged to 25 weeks. Using a × 40 objective, confocal z-stacks were taken through the RGC layer in three consistent regions in both central and peripheral retina. RGCs were counted manually using *Cell counter* available in AnalySIS Lifescience software (version 2.8, build 1235; Olympus Soft Imaging Solutions, Germany) and are presented as the number of cells/mm^2^ of retina.

To evaluate RGC dendritic morphology, z-stack images of three GFP^+^ RGCs per mouse (n = 3 mice/genotype/age; 3, 6, 12 and 20 weeks old) were obtained using a 20 × objective to capture the entire dendritic tree. Images were imported into Fiji open-source image processing software [36] and dendritic morphology was assessed manually using plug-ins. A-type RGCs were identified based on morphological characteristics i.e. soma size, dendritic field size and the radiating pattern of branching as previously described [38]. Dendritic tree area was measured using the convex polygon tool to connect the distal tips of dendrites to form a polygon. Total dendritic length was determined using *NeuronJ* available in Fiji. and the total number of branches/nodes was determined using *Cell counter*. All analyses were performed by a user blind to mouse genotype and age.

### Mass spectrometry to quantitate stored heparan sulphate

Unfixed brain slices, optic nerve and retina were homogenised in 0.02 M Tris/0.5 M NaCl, pH 7.4, with HS disaccharides quantified using a previously published method [41]. Peak area ratios were determined using Analyst 1.6.2 software (ABSciex, Concord, Ontario, Canada), expressed per mg of total protein.

### Statistics

Data are expressed as mean ± SEM or as individual data points. Graphical and analytical exploratory data analyses were performed, and patterns assessed. To compare differences between unaffected and MPS IIIA mice at each time point, an un-paired Student’s *t*-test with a Welch’s correction was utilised. (GraphPad Prism version 8). Regression models with continuous scale outcome variables and time-dependant interaction effects were fitted to the data, and residuals analysed to validate the models’ distributional assumptions. Multiple comparisons were handled with ANOVA and post-hoc Bonferroni correction. Data were regarded as statistically significant when *p* < 0.05.

## Results

**Fig. 1:**
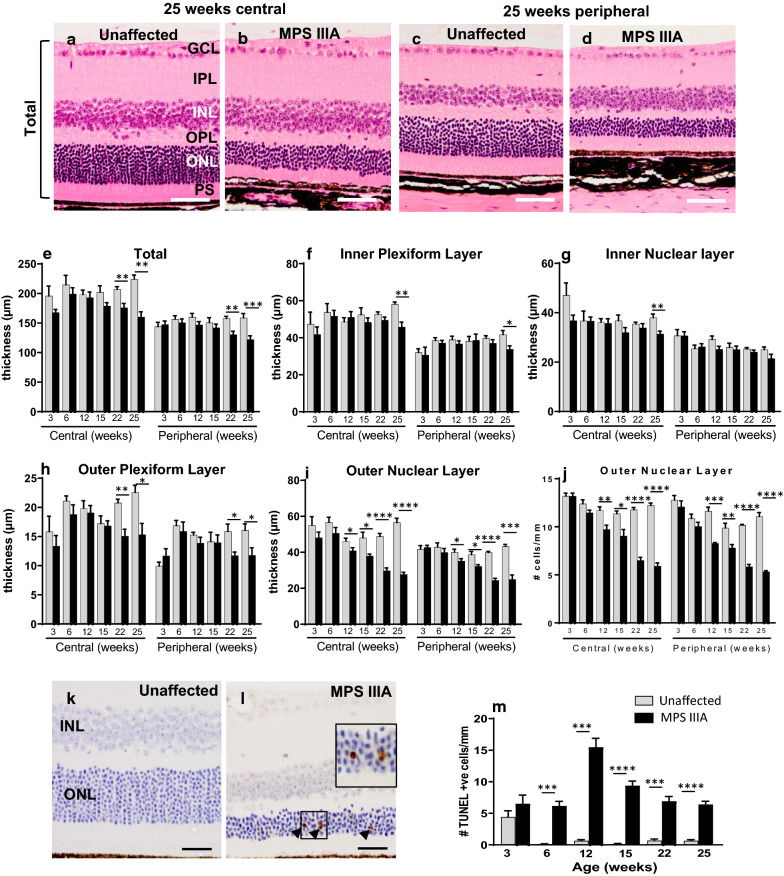
Evaluation of retinal thickness in three to 25 week old unaffected and MPS IIIA mice. Representative images of haematoxylin and eosin-stained retina from 25 week old unaffected and MPS IIIA mice are shown. Photos show central (**a**, **b**) and peripheral (**c**, **d**) retina. **e** Total retinal thickness, defined as the distance from the ganglion cell layer (GCL) to the photoreceptor segments (PS), was quantified in mice aged 3–25 weeks. The thickness of the inner plexiform layer (IPL; **f**), inner nuclear layer (INL; **g**) and outer plexiform layer (OPL; **h**) was determined. The thickness of (**i**) and number of rows of cells in (**j**) the outer nuclear layer (ONL) is shown. Representative images of TUNEL labelling in 25 week old unaffected (**k**) and MPS IIIA (**l**) mouse central retina. **m** Quantification of TUNEL-positive cells in the retina of unaffected and MPS IIIA mice aged three to 25 weeks. Scale bar: **a**–**d** = 50 µm; **k**, **l** = 25 µm. Data are expressed as mean ± SEM. *p < 0.05, **p < 0.01, ***p < 0.001, ****p < 0.0001

Figure 1a–d shows representative photos of H&E-stained 25 week old unaffected and MPS IIIA mouse retinae. Total retinal thickness declined progressively in MPS IIIA mice (Fig. 1e). We observed no change in the thickness of the IPL (Fig. 1f) or INL (Fig. 1g) until MPS IIIA mice reached 25 weeks of age, however the OPL (Fig. 1h) exhibited thinning from 22 weeks of age, and the ONL (Fig. 1i) was notably thinner in affected mice from ~ 12 weeks of age, a finding replicated when the number of photoreceptor cells was counted (Fig. 1j). TUNEL-positive, presumptively apoptosing cells were observed in the MPS IIIA mouse ONL, from 6 weeks of age with the peak period occurring around 12 weeks of age. (Fig. 1k–m).

**Fig. 2:**
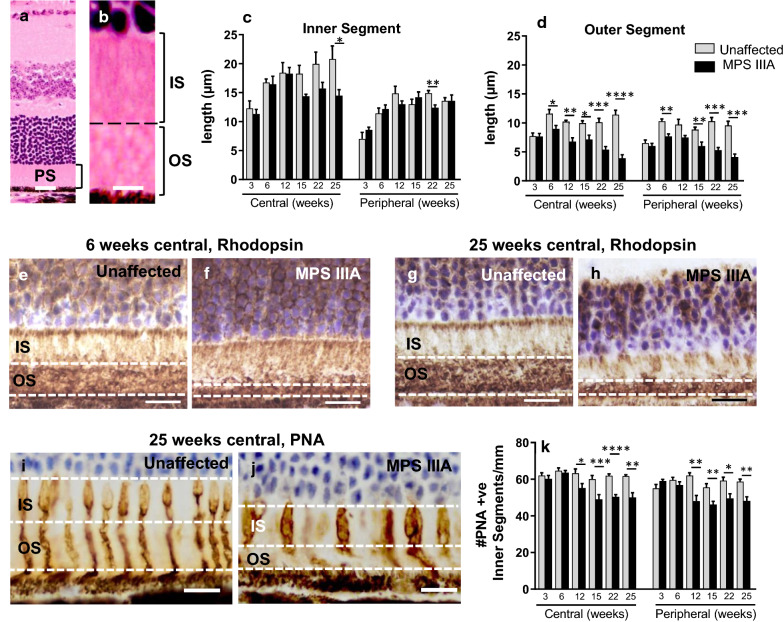
Evaluation of the inner and outer segments in three to 25 week old unaffected and MPS IIIA mice. Haematoxylin and eosin-stained retina showing the photoreceptor segment (PS) layer (**a**), magnified in (**b**) to delineate inner (IS) and outer (OS) segments. Quantification of inner (**c**) and outer (**d**) segment length in central and peripheral retina. **e**–**h** Representative images of rhodopsin immunostaining detailing rod outer segments in 6 and 25 week old unaffected and MPS IIIA mouse central retina. Peanut agglutin (PNA)-stained cone segments in 25 week old unaffected (**i**) and MPS IIIA (**j**) central retina. **k** Quantification of the number of PNA-stained inner segments. Scale bar; **a** = 25 µm; **b** = 5 µm; **e**–**h** = 20 µm; **i**, **j** = 10 µm. Data are expressed as mean ± SEM. *p < 0.05, **p < 0.01, ***p < 0.001, ****p < 0.0001

Examining the ONL more closely (Fig. 2a, b) we noted fewer outer segments in both regions of retina in MPS IIIA mice as early as 6 weeks of age (Fig. 2d). Staining of rod photoreceptors with rhodopsin (Fig. 2e–h) and cone photoreceptors with peanut agglutinin (Fig. 2i–k) confirmed the early loss of both photoreceptor subtypes in affected mice. Thickening of the inner segments was noted in MPS IIIA mouse c.f. unaffected mouse retina (Fig. 2j).

**Fig. 3:**
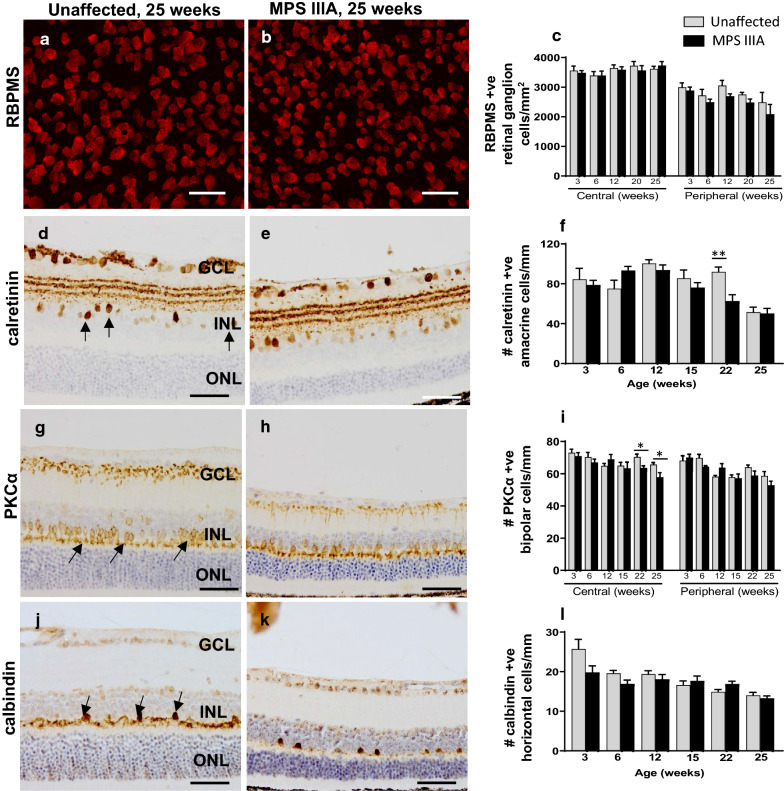
Evaluation of retinal ganglion cells, amacrine cells, bipolar cells and horizontal cells in three to 25 week old unaffected and MPS IIIA mouse retina. Representative images of RBPMS-immunolabelled retinal ganglion cells (**a**, **b**), calretinin-immunolabelled amacrine cells (**d**, **e**), PKCα-immunolabelled rod bipolar cells (**g**, **h**) and calbindin-immunolabelled horizontal cells (**j**, **k**) are shown. Arrows indicate examples of the cell type quantified. Quantification of retinal ganglion cells (**c**), amacrine cells (**f**), rod bipolar cells (**i**) and horizontal cells (**l**). Scale bar; **a**, **b** = 25 µm; **d**, **e**, **g**, **h**, **j**, **k** = 50 µm. Data are expressed as mean ± SEM. GCL, ganglion cell layer; INL, inner nuclear layer; ONL, the outer nuclear layer. *p < 0.05, **p < 0.01

In contrast, there was no significant change in the number of RBPMS-positive retinal ganglion cells (RGCs) in MPS IIIA retina to 25 weeks of age (Fig. 3a–c). The neurons had a soma diameter range of 17.8–31.5 μm and a dendritic field diameter ranging from 189.2 to 468.8 μm, consistent with previous studies [34, 38]. To determine whether more discrete changes were present, we evaluated dendritic complexity (Additional file 1: Fig. S1A, B), a measure previously used to assess neuronal responses to injury, disease, or environmental conditions [2]. Overall, RGCs in the MPS IIIA mouse retina were found to retain their characteristic morphology and fine dendritic geometry and appeared largely unaffected by the disease process at least to 20 weeks of age (Additional file 1: Fig. S1C–F). Further, there was no convincing loss of calretinin-positive amacrine cells (Fig. 3d–f) in MPS IIIA retina; the number of cells declined in mice of both genotypes with age. PKC-α-labelled rod bipolar cells (Fig. 3g–i) and calbindin-positive horizontal cells (Fig. 3j–l) also appeared largely resilient to the disease process.

**Fig. 4:**
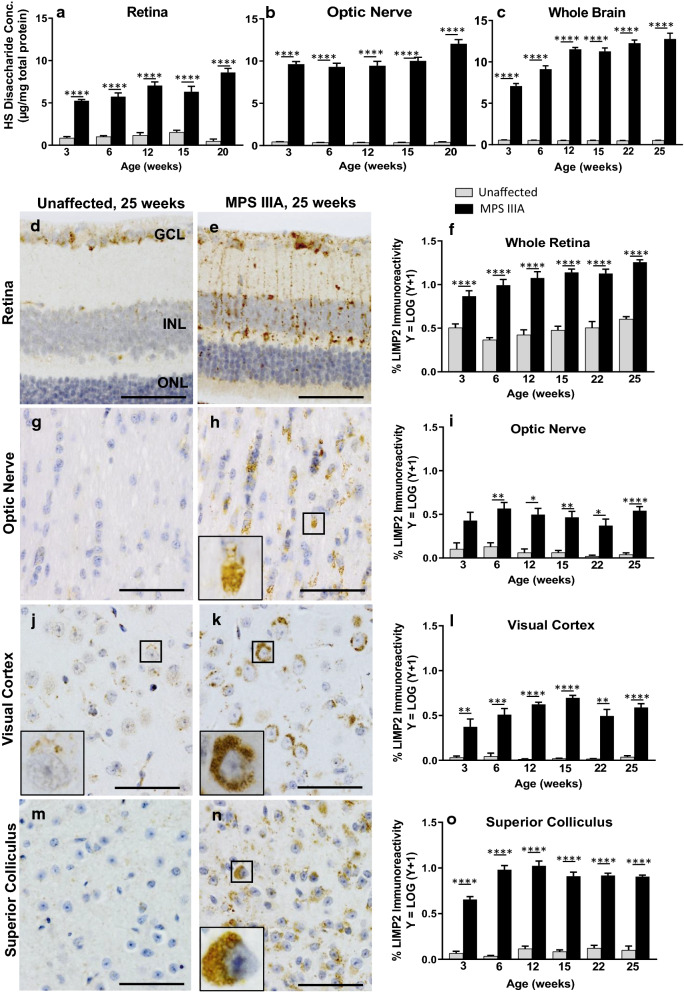
Analysis of endo/lysosomal compartment expansion in unaffected and MPS IIIA mouse retina, optic nerve, and brain. Quantification of stored heparan sulphate in the retina (**a**), optic nerve (**b**) and whole brain (**c**) in three to 25 week old mice. Representative images of LIMP2 immunolabelling in 25 week old unaffected and MPS IIIA mouse retina (**d**, **e**), optic nerve (**g**, **h**), visual cortex (**j**, **k**) and superior colliculus (**m**, **n**). Quantification of % LIMP2 immunoreactivity in the retina (**f**), optic nerve (**i**), visual cortex (**l**) and superior colliculus (**o**). Scale bar; **d**, **e**, **g**, **h**, **j**, **k**, **m**, **n** = 50 µm. Data are expressed as mean ± SEM. *p < 0.05, **p < 0.01, ***p < 0.001, ****p < 0.0001

We next quantified HS levels in retina, optic nerve and brain (Fig. 4a–c), and to confirm and visualise the impact of substrate accumulation, stained the tissues with a marker of the late endosome/lysosome compartment (lysosomal integral membrane protein, LIMP 2; Fig. 4d–o). Significant and progressive accumulation of HS occurred in MPS IIIA mouse retina, optic nerve and brain from 3 weeks of age (c.f. levels in unaffected mouse tissues). Elevated LIMP 2 expression was also evident in retina, optic nerve and brain (staining quantified in the visual cortex and superior colliculus; Fig. 4f, i, l, o) from 3 weeks of age and the amount of immunoreactivity increased to 25 weeks of age in MPS IIIA mice.

**Fig. 5:**
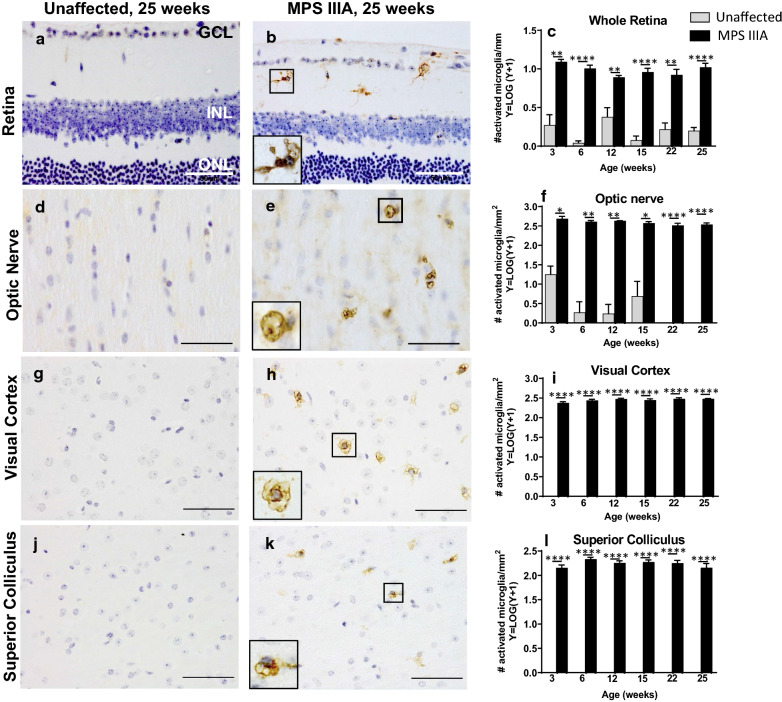
Evaluation of microglial activation in three to 25 week old unaffected and MPS IIIA mouse retina, optic nerve and brain. Representative images of isolectin-B4 staining in 25 week old unaffected and MPS IIIA mouse retina (**a**, **b**), optic nerve (**d**, **e**), visual cortex (**g**, **h**) and superior colliculus (**j**, **k**). Insets show higher-power images of ameboid, presumptively activated microglial cells in MPS IIIA mouse tissues. Scale bar = 50 µm. Quantification of activated microglia in the retina (**c**), optic nerve (**f**), visual cortex (**i**) and superior colliculus (**l**) revealed elevated numbers of activated microglia in all tissues in MPS IIIA mice from 3 weeks of age. Data are mean ± SEM. *p < 0.05, **p < 0.01, ****p < 0.0001

**Fig. 6:**
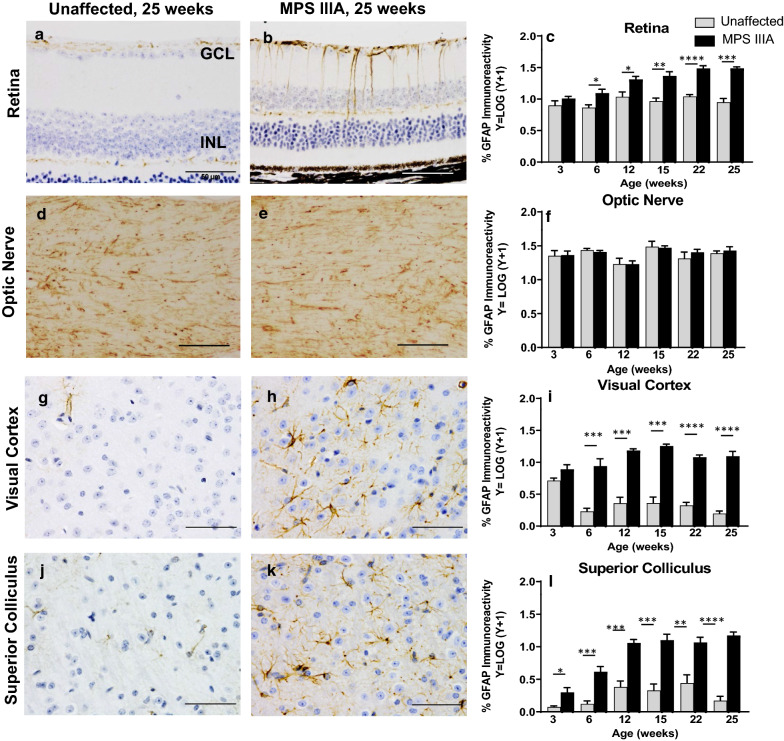
Analysis of GFAP-immunoreactivity in three to 25 week old unaffected and MPS IIIA mouse retina, optic nerve and brain. Representative images of GFAP immunolabelling in 25 week old unaffected and MPS IIIA mouse retina (**a**, **b**), optic nerve (**d**, **e**), visual cortex (**g**, **h**) and superior colliculus (**j**, **k**). Scale bar = 50 µm. Quantification of % GFAP immunoreactivity in the retina (**c**), optic nerve (**f**) visual cortex (**i**) and superior colliculus (**l**) revealed elevated GFAP expression in all MPS IIIA tissues except optic nerve. Data are mean ± SEM. *p < 0.05, **p < 0.01, ***p < 0.001, ****p < 0.0001

Amoeboid, isolectin-B4-positive and therefore presumptively activated microglia were noted in MPS IIIA mouse retina and optic nerve, as well as visual cortex and superior colliculus from 3 weeks of age, the earliest time point evaluated (Fig. 5a–l). A cumulative increase in retinal and brain GFAP expression was noted in affected mice to 25 weeks of age (Fig. 6a–c, g–l); however, no change was observed in optic nerve (Fig. 6d–f).

Brain tissue from MPS IIIA mice exhibits increasing numbers of ubiquitin-positive axonal inclusions with age [4]. Whilst retina contained exceedingly few ubiquitin-positive structures (data not shown), axons in MPS IIIA mouse optic nerve exhibited immunoreactive spheroids that increased in number as the disease progressed (Additional file 2: Fig. S2A–C).

**Fig. 7:**
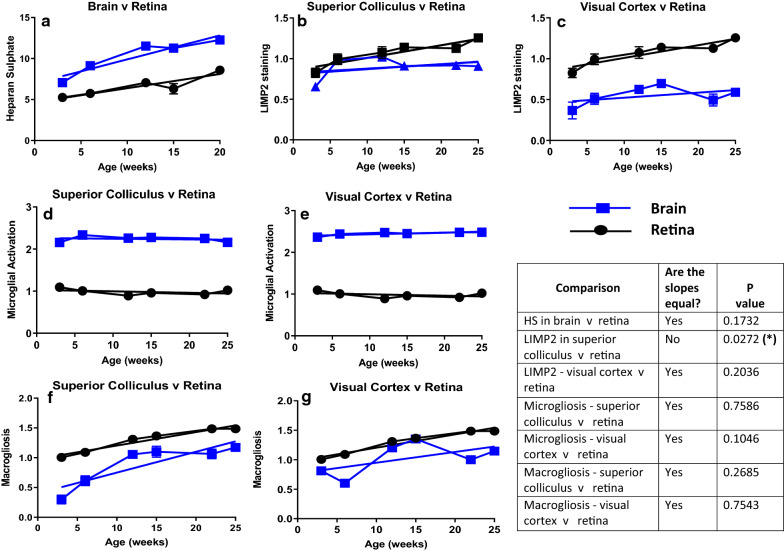
Relationship between the age of onset and rate of accumulation of disease lesions in the MPS IIIA mouse brain (squares) and retina (circles). Data indicating enlargement of the LIMP2-positive endo/lysosomal compartment (**a**–**c**), presence of activated microglia (**d**, **e**) and elevated expression of GFAP by macroglia (**f**, **g**) is shown. The slope of the lines was compared, and *p* values have been tabulated

For retinal imaging to provide a non-invasive, quantitative method for visualising neurodegenerative changes in Sanfilippo syndrome, retinal disease must parallel brain disease. When we compared the rate of accumulation of HS (Fig. 7a), the rate of endo/lysosomal expansion (Fig. 7b, c), the appearance of activated microglia (Fig. 7d, e) or the number of GFAP-reactive macroglia (Fig. 7f, g) in MPS IIIA mouse brain to that in retina across the time-frame of the study, we found little difference between the two tissue types. LIMP2 accumulated at a statistically significantly slower rate in superior colliculus c.f. retina, but there was no difference in the rate of LIMP2 accumulation in visual cortex c.f. retina. Nor was there a difference in the rate of development of any of the other disease lesions in brain versus retina, with all other slopes found to be not significantly different to each other, suggesting the rate of disease lesion progression is equivalent in the two CNS tissue types.

**Fig. 8:**
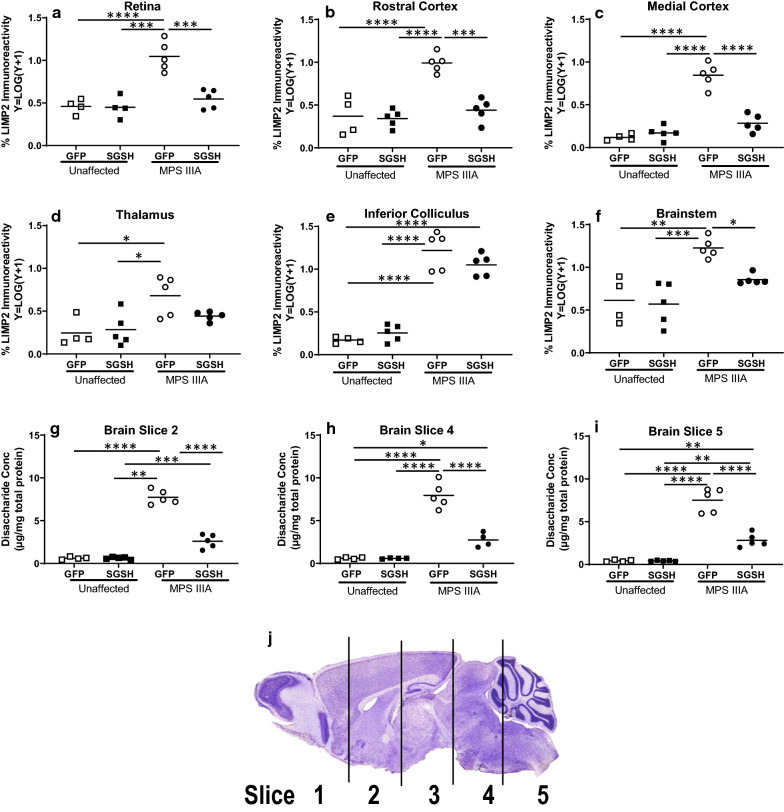
AAV9-CMV-SGSH (or control vector) was delivered i.v. to mice on each of day 0 and day 5 of life and the impact on substrate accumulation in the endo/lysosomal compartment in mouse retina and brain was determined 6 weeks post-injection. % LIMP2 immunoreactivity was evaluated in the retina (**a**), rostral cortex (**b**), medial cortex (**c**), thalamus (**d**), inferior colliculus (**e**) and brainstem (**f**). Accumulation of primary substrate (heparan sulphate) in brain slice 2, 4 and 5 is shown in **g**–**i**). The 2 mm thick brain slice protocol is shown in **j**. *p < 0.05, **p < 0.01, ***p < 0.001, ****p < 0.0001

Finally, given there is currently no effective therapy for patients with Sanfilippo syndrome, and any treatment strategy developed needs to be capable of preventing development of both brain and retinal disease lesions to afford patients maximum quality of life, we examined the short-term efficacy of i.v. delivery of AAV9-CMV-hSGSH to MPS IIIA mice at day 0 and 5 of life. One unaffected control mouse was cannibalised between day 0 and day 5 of life, however all other mice remained healthy post-injection. Mouse retina (Fig. 8a) and brain (Fig. 8b–f) was evaluated at 6 weeks post-injection. AAV9-CMV-hSGSH normalised the size of the endo/lysosomal compartment in MPS IIIA mouse retina and also in some (thalamus, rostral and medial aspects of cerebral cortex) but not all (inferior colliculus and brainstem) areas of brain examined and significantly reduced HS levels in brain (Fig. 8g–i).

## Discussion

Our goal was to establish the age at which disease lesions first become apparent in Sanfilippo mouse retina and brain and compare the rate of disease progression in each tissue in order to test the hypothesis that examination of the retina will provide insight into brain disease stage. In short-term studies, we then determined the ability of a clinically-relevant gene therapy approach to mediate improvements in disease sequelae in both neural tissues. Our objective is to ultimately provide a therapeutic monitoring strategy in addition to a prognostic tool, for children diagnosed with this devastating disorder. Whilst a few researchers have evaluated disease lesions in MPS III mouse retina, those studies have either started later in the disease course (three-plus months of age [21, 42]) or were qualitative in nature [18]. None have sought to compare brain and retinal disease with the goal of facilitating non-invasive evaluation of retina in order to inform understanding of brain disease.

For the first time, our study reveals that the age of onset and rate of progression of retinal disease in mice with MPS IIIA are remarkably similar to those in the brain. Both tissues exhibit substantial accumulation of heparan sulphate by 3 weeks of age, as determined by sensitive mass spectrometry-based measurements. HS levels in optic nerve were also elevated at this pre-symptomatic age. Primary storage of HS led to massive expansion of the late endosomal/lysosomal system in retina, optic nerve and brain as visualised using LIMP2-staining. Similarly, the presence of presumptively activated isolectin-B4-positive microglia with an amoeboid morphology was noted in all three tissues in 3-week-old MPS IIIA mice. These cells remained present to the end of the study (mice aged 25 weeks) and were only rarely observed in unaffected mouse retina and optic nerve. Macrogliosis was observed from ~ 6 weeks of age in MPS IIIA mouse retina and brain although curiously, not in optic nerve. This may be explained by the fact that the optic nerve inherently expresses a high level of GFAP—astrocytic processes extend in all directions and through all planes of the optic nerve, rendering astrogliosis more challenging to quantify. Staining of axons in optic nerve with antibodies to ubiquitin revealed the appearance of axonal spheroids between 3 and 6 weeks of age, an observation that has also been made in brain [17]. Thus, the age of onset of disease lesions in MPS IIIA mouse retina and brain is the same: ~ 3 weeks of age.

Disease lesion progression also occurs in parallel in the two structures. This suggests that methods that visualise and quantitate any of these lesions in MPS IIIA retina could inform clinicians about brain disease progression. Similarly, the efficacy of any treatment that is able to access retina as well as brain, for example i.v. AAV9-sulphamidase as examined in the present study, or small molecule therapeutics such as anti-inflammatory agents e.g. Anakinra; IL-1R inhibitor, which is currently under clinical evaluation in Sanfilippo patients (www.clinicaltrials.gov; NCT04018755), could ostensibly be visualised using non-invasive retinal imaging.

The possibility of using retinal lesions to inform our understanding of the degree of brain pathology is receiving increasing attention from researchers evaluating a range of neurodegenerative diseases, most notably Alzheimer’s disease (AD). Koronyo-Hamaoui et al. [22] were first to describe the presence of Aβ + plaques in AD patient retinae, establishing a curcumin-based imaging strategy to facilitate the early diagnosis and prognosis of AD patients. This technique has recently been evaluated in humans with this disorder [23]. Tau oligomers are also present in retina early in the disease process [30], and they are associated with neuroinflammatory cells, a phenomenon also noted in brain. Similarly, work on the 3xTg model of AD indicated that tau tangles in addition to Aβ plaques and gliosis occurs in the RGL pre-symptomatically and could potentially be used to ‘predict’ brain pathology in AD [14].

More recent studies demonstrate that hyperspectral imaging enables brain-relevant disease lesions to be evaluated in retina [15]. Changes in retinal reflectance spectra were found to correlate with brain Aβ load in patients with mild cognitive impairment. Similar observations were made in 5xFAD mice, suggesting that retinal hyperspectral imaging may be a useful tool for predicting brain Aβ load [15]. Responding to the rapidly expanding body of research linking ocular pathology and AD brain lesions, the American Alzheimer’s Association convened a ‘think tank’ in mid-2019 aimed at establishing the foundations for population-based detection and monitoring of mild cognitive impairment and AD [37]. Other disorders for which retinal imaging is being explored as a screening/prognostic tool include Parkinson’s disease [20], psychiatric diseases including schizophrenia, bipolar disorder and major depressive disorder [39] and multiple sclerosis [25].

The second novel outcome of our study is the characterisation of retinal lesions present very early in the MPS III disease course, i.e. pre-symptomatically. Other studies have only evaluated Sanfilippo retina from a symptomatic stage onwards (12 + weeks [21, 42]). We observed apoptotic (TUNEL-positive) cells and shortening of photoreceptor outer segments in both central and peripheral retina from 6 weeks of age, followed by loss of inner segments and profound, progressive loss of photoreceptor cell bodies 12 weeks of age in MPS IIIA mice. Both rod and PNA^+^-cone photoreceptor cells are impacted. Tools capable of visualising these very early apoptotic events and/or detecting initial loss of outer segments may provide prognostic indicators of the early-onset form of Sanfilippo c.f. later-onset disease that manifests in adulthood [29].

Other than photoreceptors, all other neuronal classes in the retina, including RGCs, bipolar cells, horizontal cells and amacrine cells, appear largely unaffected by the disease process. These parallels observations made in MPS IIIA mouse brain tissue: neither we [4], nor others [44] have been able to document neuron loss to very late disease stages (9 months of age). Therefore, apart from photoreceptors which must be acknowledged to be a highly specialised class of neuron, all other neurons in the retina and brain remain intact throughout the symptomatic stages of the disease process. Our observations are compatible with those reported in MPS IIIB mice by Heldermon et al. [18], who showed visible thinning of the ONL between eight and 16 weeks of age, and with those of Intartaglia et al. [21] who have recently described ONL thickness reductions and loss of photoreceptor outer segments at later stages in MPS IIIA mice (~ 6 months of age) and likewise reported minimal involvement of other retinal cell types.

Loss of rod photoreceptors presumptively leads to the impairments previously noted in the dark-adapted ERG in MPS III mice [18, 21, 42]. Impaired or absent scotopic responses have also been described in patients with MPS III who exhibited night blindness [13]. Abnormalities in light-adapted ERGs were not noted in MPS IIIB mice aged up to 40 weeks of age [18] but became apparent by ~ 1 year [42]. The mechanism underlying photoreceptor cell death is at present unknown but may relate to impaired autophagosome/lysosome fusion which reduces autophagic flux [21].

In conclusion, this is the first study to evaluate the relationship between retinal and brain disease lesions in an animal model of a lysosomal storage disorder. We demonstrate that both retina and brain in Sanfilippo mice exhibit disease lesions at a very early disease stage (pre-symptomatic; 3 weeks of age) and that the rate of disease progression is comparable between the two structures. This bodes well for utilising non-invasive imaging of retina to inform our understanding of the state of brain disease. The loss of photoreceptor neurons during early disease progression and the possibility of visualising and monitoring this using non-invasive techniques may provide both a prognostic and therapeutic monitoring tool for MPS IIIA. At the very least, the early age at which retina displays disease lesions in Sanfilippo syndrome highlights the absolute necessity of directing therapeutics at both the brain and the retina, to ensure patient quality of life is maximised.

## Supplementary information

**Additional file 1: Figure S1.** A-type retinal ganglion cells in unaffected Thy1-GFP (A) and MPS IIIA Thy1-GFP mouse retina (B). Scale bar = 50 μm. The complexity of the dendritic tree was determined in GFP-positive RGCs in unaffected and MPS IIIA mice aged three, six, 12, and 20 weeks of age. We observed no significant difference in dendritic tree area (C), total dendritic tree cable length (D), or the total number of branch points or nodes (E). Negligible change was observed in soma diameter over the 20 week time course (F). Data represent mean ± SEM. *p<0.05.

**Additional file 2: Figure S2.** Representative images of ubiquitin immunolabelling in 25 week old unaffected and MPS IIIA optic nerve are shown (A, B). Arrows indicate ubiquitin-positive spheroids. Scale bar = 20 µm. (C) Ubiquitin-positive axonal spheroids >5 µm diameter were quantified in optic nerve obtained from unaffected and MPS IIIA mice aged 3- to 25-weeks of age. ****p<0.0001.

## Data Availability

The datasets created during the current study are available from the corresponding author on reasonable request. No publicly available data was used in the manuscript.
