# Supplementary figures and images for: Is the eye a window to the brain in Sanfilippo syndrome?
Source: Acta Neuropathol Commun. 2020 Nov 17;8:194. doi: 10.1186/s40478-020-01070-w (PMC7672954; doi:10.1186/s40478-020-01070-w)

Supplementary Figure 1

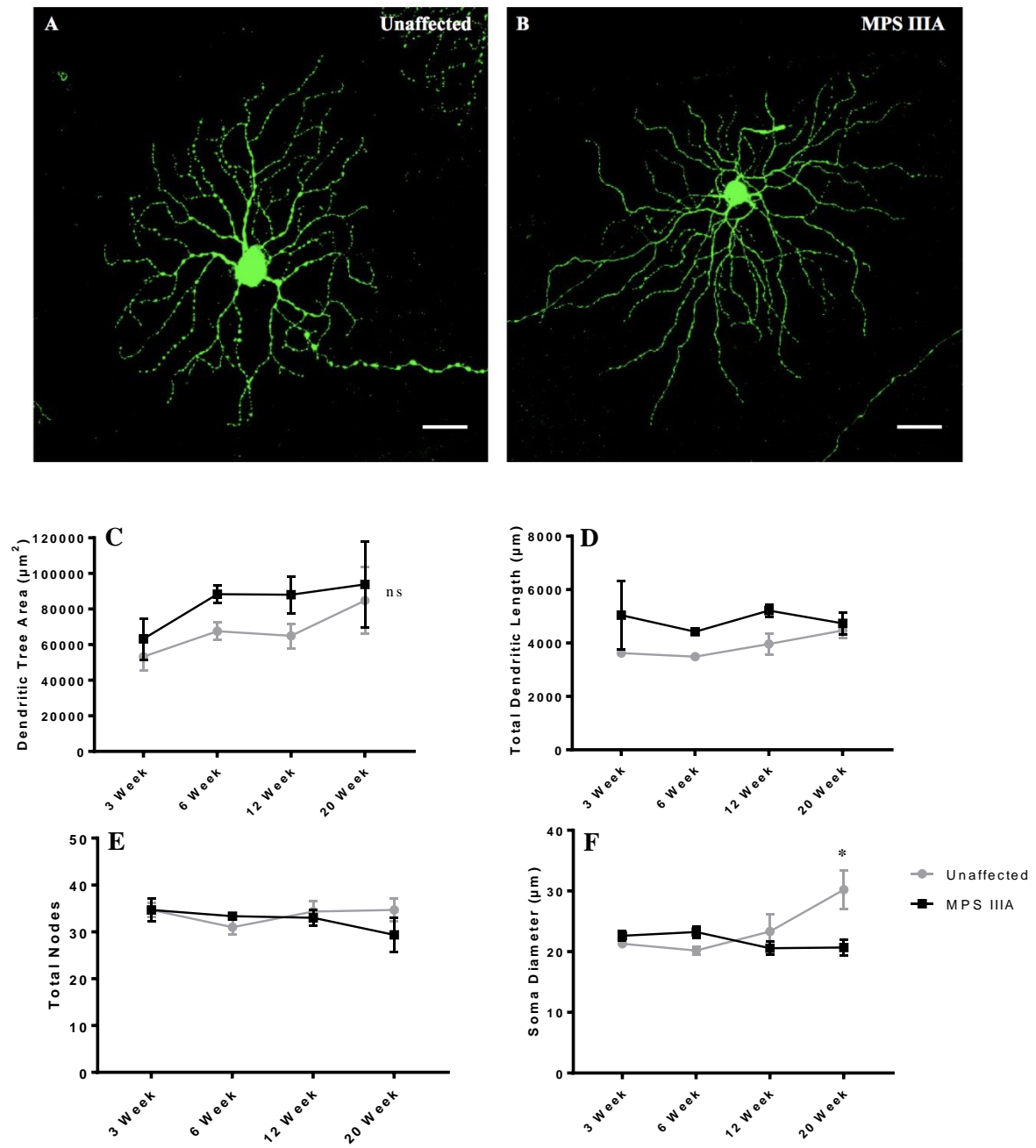

Supplement: Supplementary file 1 — Additional file 1: Figure S1. A-type retinal ganglion cells in unaffected Thy1-GFP (A) and MPS IIIA Thy1-GFP mouse retina (B). Scale bar = 50 μm. The complexity of the dendritic tree was determined in GFP-positive RGCs in unaffected and MPS IIIA mice aged three, six, 12, and 20 weeks of age. We observed no significant difference in dendritic tree area (C), total dendritic tree cable length (D), or the total number of branch points or nodes (E). Negligible change was observed in soma diameter over the 20 week time course (F). Data represent mean ± SEM. *p<0.05. [file 40478_2020_1070_MOESM1_ESM.pdf]

Supplementary Fig. 2

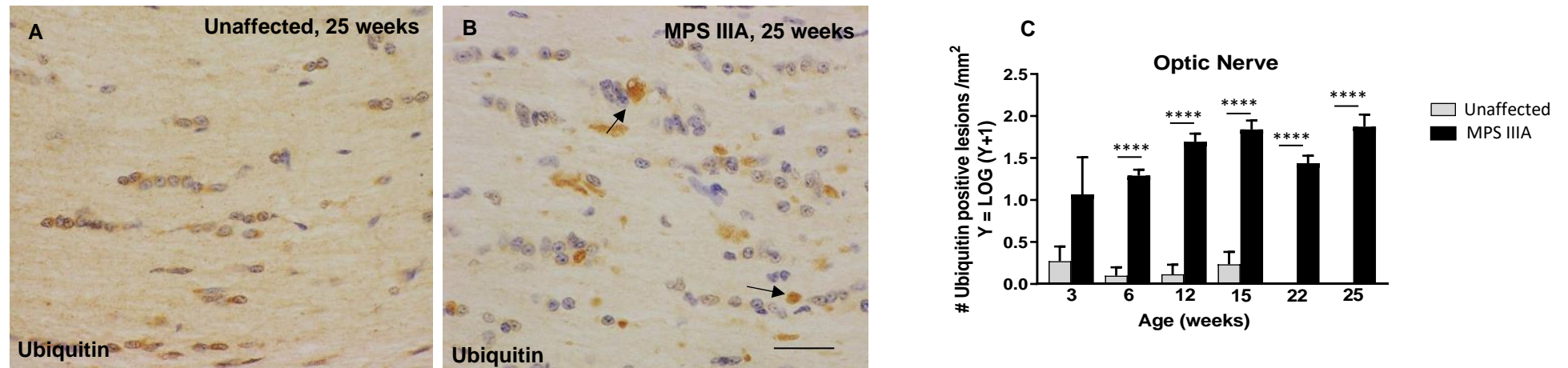

Supplement: Supplementary file 2 — Additional file 2: Figure S2. Representative images of ubiquitin immunolabelling in 25 week old unaffected and MPS IIIA optic nerve are shown (A, B). Arrows indicate ubiquitin-positive spheroids. Scale bar = 20 µm. (C) Ubiquitin-positive axonal spheroids >5 µm diameter were quantified in optic nerve obtained from unaffected and MPS IIIA mice aged 3- to 25-weeks of age. ****p<0.0001. [file 40478_2020_1070_MOESM2_ESM.pdf]
